# Characterization of Porous β-Type Tricalcium Phosphate Ceramics Formed via Physical Foaming with Freeze-Drying

**DOI:** 10.3390/ijms25105363

**Published:** 2024-05-14

**Authors:** Kazuaki Hashimoto, Hiroto Oikawa, Hirobumi Shibata

**Affiliations:** Department of Applied Chemistry, Faculty of Engineering, Chiba Institute of Technology, 2-17-1 Tsudanuma, Narashino-shi 275-0016, Chiba, Japan; oikawa6et0@gmail.com (H.O.); hirobumi.shibata@p.chibakoudai.jp (H.S.)

**Keywords:** porous β-tricalcium phosphate, physical foaming method, freeze-drying, cellulose nanofiber, nonionic surfactants, hydrophilic/lipophilic balance (HLB)

## Abstract

Porous β-tricalcium phosphate (Ca_3_(PO_4_)_2_; β-TCP) was prepared via freeze-drying and the effects of this process on pore shapes and sizes were investigated. Various samples were prepared by freezing β-TCP slurries above a liquid nitrogen surface at −180 °C with subsequent immersion in liquid nitrogen at −196 °C. These materials were then dried under reduced pressure in a freeze-dryer, after which they were sintered with heating. Compared with conventional heat-based drying, the resulting pores were more spherical, which increased both the mechanical strength and porosity of the β-TCP. These materials had a wide range of pore sizes from 50 to 200 µm, with the mean and median values both approximately 100 µm regardless of the freeze-drying conditions. Mercury porosimetry data showed that the samples contained small, interconnected pores with sizes of 1.24 ± 0.25 µm and macroscopic, interconnected pores of 25.8 ± 4.7 µm in size. The effects of nonionic surfactants having different hydrophilic/lipophilic balance (HLB) values on foaming and pore size were also investigated. Materials made with surfactants having lower HLB values exhibited smaller pores and lower porosity, whereas higher HLB surfactants gave higher porosity and slightly larger macropores. Even so, the pore diameter could not be readily controlled solely by adjusting the HLB value. The findings of this work indicated that high porosity (>75%) and good compressive strength (>2 MPa) can both be obtained in the same porous material and that foaming agents with HLB values between 12.0 and 13.5 were optimal.

## 1. Introduction

A wide variety of synthetic materials, including metals, polymers, and ceramics, are currently being investigated with regard to clinical applications as bone substitutes [1,2,3,4]. Of particular interest are sintered calcium phosphates, especially hydroxyapatite ceramics (Ca_10_(PO_4_)_6_(OH)_2_; HAp) [5,6], β-TCP [5,7], and biphasic calcium phosphate ceramic composites (BCP) [8,9,10]. This interest is primarily related to the fact that bone is largely composed of calcium phosphate apatite minerals. HAp implants are more crystalline than biological bone and so these implants exhibit greater chemical stability in vivo and tend not to degrade after implantation [11,12]. In the case that resorption does not occur in such implants, bone deformation [13] and the risk of fracture around the replacement bone may increase in the long term [11]. In contrast to HAp ceramics, β-TCP ceramic implants are bioabsorbable and easily replaced with new autogenous bone [14,15]. Because the solubility of these materials is close to that of living bone, these ceramics are also not soluble under physiological conditions (meaning a pH of 7.4 and temperature of 37 °C) [16] and instead are typically absorbed by osteoclasts. The associated mechanism is believed to involve local acidification, leading to the dissolution of the sintered β-TCP [14,17]. In addition to this osteoconductive capacity, sintered β-TCP has been shown to exhibit significant osteoinductive characteristics [18]. For these reasons, sintered β-TCP is one of the most attractive bone replacement materials. In particular, porous β-TCP can be used as a scaffold material to promote the repair of bone tissue because it has suitable pore sizes to promote the growth of bone tissue within the porous material [19,20]. β-TCP also tends to contain macropores (100–600 µm) together with micropores (0.1–5 µm) [21] and connections between these pores allow nutrients, blood, cells, and biological tissue to enter, thereby promoting autogenous ossification. Many previous studies have demonstrated these effects [22,23,24,25,26,27].

Porous bodies can be produced with relative ease using foaming methods [28,29], in which a foaming agent is added and ambient air is entrained via mechanical agitation. In these techniques, the foam is typically stabilized by adding a surfactant based on the Gibbs–Marangoni effect, in which surfactant molecules compensate for surface tension gradients [30]. Prior research by the authors has demonstrated that porous materials prepared u foaming methods exhibit biases in pore size and pore distribution. This bias occurs due to various factors involved in the formation of bubbles, such as flow due to gravity, surface viscosity, the contribution of capillary attraction at the plateau boundary to membrane liquid drainage, and gas diffusion associated with the coalescence and redistribution of bubbles [31]. An especially important factor is the capillary attraction at the plateau boundary, at which bubbles are adjacent to one another. The rate of flow to the plateau boundary has been shown to be correlated with the viscosity of the adsorption layer near the surface. It has also been reported that a higher viscosity will increase the thickness of the film between bubbles such that the bubble lifetime is increased [32]. The Young–Laplace equation [33], describing gas diffusion, states that, in the case that two bubbles of different sizes come into contact with one another, the air from the smaller bubble (which has a higher internal pressure) will diffuse through the bubble film towards the larger bubble. As a consequence, smaller bubbles are absorbed by larger bubbles, causing the bubbles to merge, and resulting in the disappearance of some bubbles.

The authors previously attempted to fabricate porous materials by adding a nonionic surfactant to β-TCP slurries as a foaming agent together with cellulose nanofibers (CNFs) as a thickening agent and bubble stabilizer. These additives were employed in conjunction with a physical foaming method in which pores were formed with mechanical stirring. This prior work showed that, in trials using a conventional foaming method, pores were uniformly distributed throughout the specimens and porous bodies with the high mechanical strength of a slurry-hardened body could be obtained [34]. It was also found that the gravity-induced outflow of water into the lower layers and the coalescence of bubbles prevented the pores from concentrating at the top of the sample and prevented the pores from coarsening [35]. However, using this method, it was challenging to properly disperse the CNFs and these fibers tended to agglomerate during drying in association with heating. This agglomeration resulted in sintered porous bodies having irregular pore shapes rather than spherical pores. In later work, porous materials were generated using CNFs, in which some of the -OH groups on the CNFs were acetylated [36] or replaced with phosphite groups [37]. The resulting porous materials were found to have spherical pores. Furthermore, we prepared porous β-TCP using a similar method, instead of CNFs with the thickening effect of carboxymethylcellulose ammonium salt, and found that porous β-TCP could be prepared with suitable pore size, porosity, and mechanical strength for bone-filling material, without any difference in pore size distribution according to the sample location [38].

In the present study, foamed β-TCP slurries were rapidly frozen and solidified, after which dried porous materials were obtained from the frozen specimens, from which water was removed via freeze-drying. This was an alternative to slower heat-based drying. The freeze-drying method used in this work was superior in that the water in the slurry was rapidly frozen at reduced pressure, after which the material was returned to room temperature such that the water evaporated without going through the liquid phase. This process allowed the samples to be dried without destroying the pore structure of the frozen slurry. The effects of varying the fabrication conditions were investigated with a focus on quick freezing. Porous specimens were also fabricated using nonionic surfactants with different hydrophilic/lipophilic balance (HLB) values [39] to control the pore size, and the physical properties of the resulting porous materials were evaluated.

## 2. Results and Discussion

**Figure 1:**
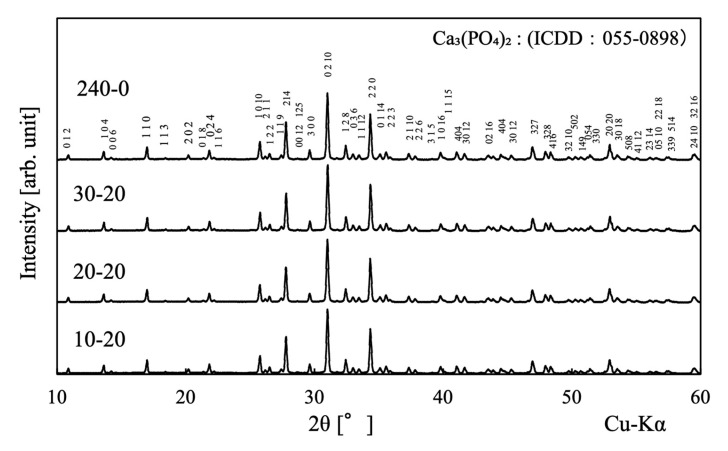
XRD patterns for porous samples fabricated in this work. The specimen names in the figure are given in Section 3.

Figure 1 presents XRD patterns obtained for sintered samples prepared with various hold and immersion times during pre-freezing. The numerical values indicate the Mille Index. The diffraction peaks generated by all samples can be attributed to β-TCP (ICDD No. 055-0898). On the other hand, a diffraction peak at 2θ = 32° was also observed, which was considered to be the strongest diffraction peak of HAp. Comparing this with the intensity of the strongest diffraction peak of β-TCP, the intensity ratio of HAp/β-TCP was at most 0.5%. This indicated that the porous β-TCP obtained in this work was almost a single phase.

**Figure 2:**
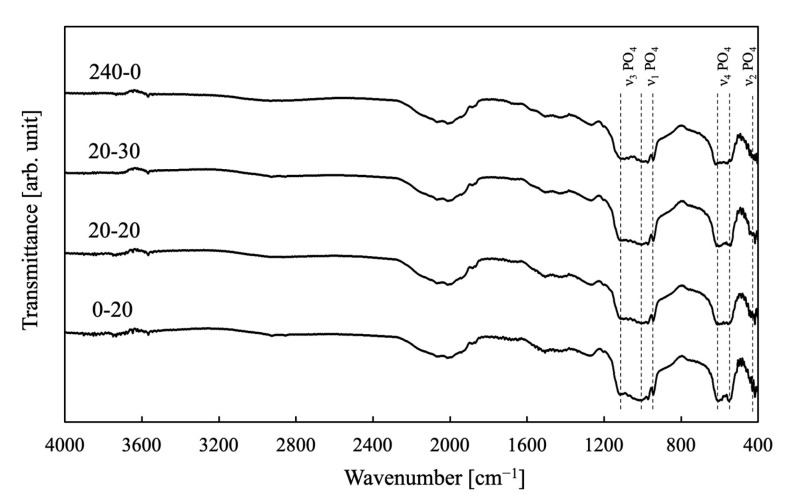
FT-IR spectra of porous samples fabricated in this work. The specimen names in the figure are given in Section 3.

Figure 2 provides the FT-IR spectra of the same samples as shown in Figure 2. These spectra exhibit bands ascribed to the bending vibration of PO_4_ groups in β-TCP at 430 cm^−1^ (ν_2_) and 560–600 cm^−1^ (ν_4_) and the stretching vibration of PO_4_ groups at 960 cm^−1^ (ν_1_), 1020 cm^−1^ (ν_3_), and 1120 cm^−1^ (ν_3_) [40]. No band attributed to P-O-P of calcium pyrophosphate was observed as a by-product, but a very small band attributed to -OH of HAp was observed at 3564 cm^−1^. This supported the results of the X-ray diffraction pattern.

**Figure 3:**
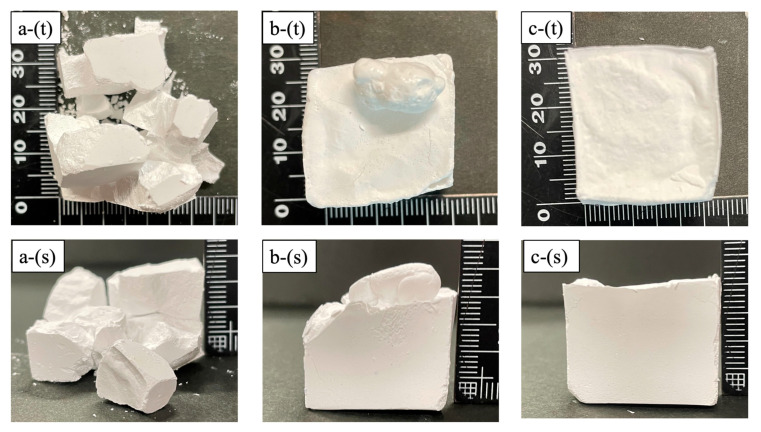
Photographic images of (**a**) 30-30, (**b**) 0-30, and (**c**) 30-20 specimens, showing (t) top view and (s) side view.

Figure 3 presents photographic images of the samples. Here, Figure 3a shows a sample (30-30) held above the liquid nitrogen surface for 30 s and immersed in liquid nitrogen for 30 s. In this case, many cracks were formed during the freezing process and it was difficult to maintain the shape of the specimen. The image in Figure 3b shows a sample (0-30) immersed in liquid nitrogen for 30 s but not held above the liquid nitrogen surface. Internal bubbles evidently exploded at the top of the sample and the original shape of the specimen was changed. These effects are attributed to the sudden temperature change caused by immersion in the liquid nitrogen. The image in Figure 3c shows a sample (30-20) held above the liquid nitrogen surface for 30 s and immersed in the liquid nitrogen for 20 s. Compared with the specimen in Figure 3a,b, the shape of the sample in Figure 3c was hardly distorted, indicating that the sample morphology could be maintained using hold and immersion times of 20 s or longer.

**Figure 4:**
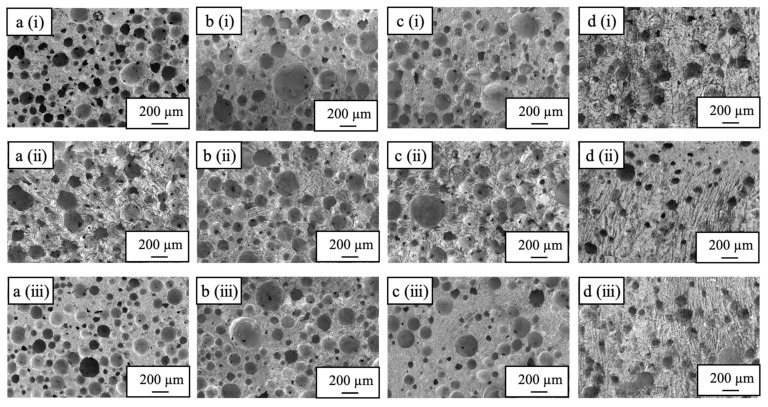
SEM images of cross-sections of (**a**) 0-10, (**b**) 20-10, (**c**) 30-10, and (**d**) 240-0 specimens, showing (**i**) top, (**ii**) center, and (**iii**) bottom sections (Secondary electron detector, high vacuum mode, accelerated voltage 5 kV).

Figure 4 shows cross-sectional SEM images of a sample immersed in the liquid nitrogen for 10 s with no hold time above the liquid nitrogen and a sample held above the liquid nitrogen for 240 s but with no immersion. The 240-0 sample exhibits spherical pores along with many crack-like pores that distort the spherical pore shapes. Holding this sample for 240 s above the liquid nitrogen caused it to freeze more slowly due to the lower thermal conductivity of the gaseous nitrogen. This, in turn, promoted the growth of ice crystals in the sample, and the sublimation of this ice during subsequent freeze-drying is thought to have produced the crack-like pores. The SEM images of the sample immersed in the liquid nitrogen for 10 s confirm the formation of spherical pores in the top and bottom parts of the sample, whereas the center part contained numerous cracks similar to those in the 240-0 specimen. These results suggest that a 10 s immersion in the liquid nitrogen was not sufficient to freeze the specimen such that subsequent cooling froze the center part.

**Figure 5:**
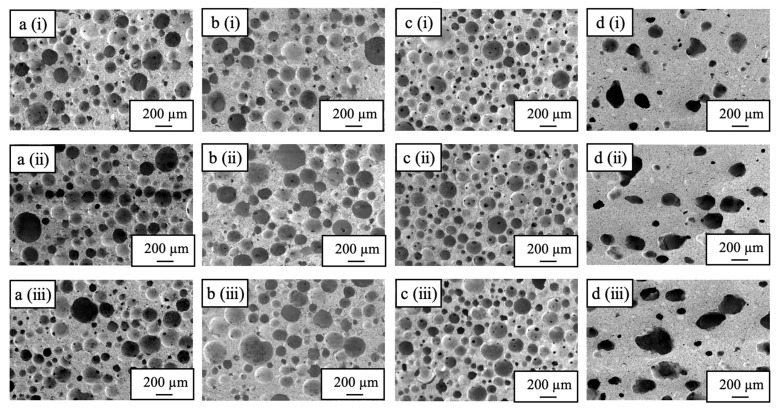
SEM images of cross-sections of (**a**) 10-20, (**b**) 20-20, (**c**) 30-20, and (**d**) heat-dried specimens, showing (**i**) top, (**ii**) center, and (**iii**) bottom sections (Secondary electron detector, high vacuum mode, accelerated voltage 5 kV).

Figure 5 presents cross-sectional SEM images of the sample immersed in the liquid nitrogen for 20 s and the samples produced with heat-drying. The latter material exhibits many distorted sphere-like pores with sizes of 200–300 µm, whereas the former sample shows spherical pores with sizes of 50–200 µm together with an interconnected pore structure. These findings indicate that the preparation of materials with spherical pores is possible based on pre-freezing with liquid nitrogen followed by freeze-drying. The sample immersed in liquid nitrogen for 20 s was evidently sufficiently frozen as no cracked pores were observed. Hence, ice crystals were not grown in this material as had occurred in the 240-0 sample.

**Figure 6:**
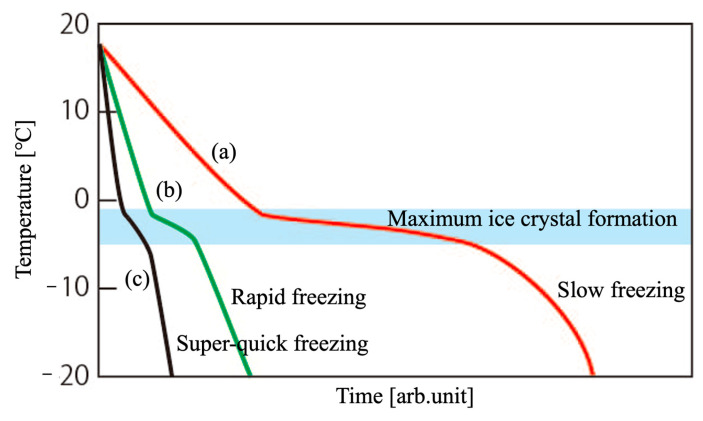
Typical time–temperature curves of water during freezing processes. (a) slow freezing, (b) rapid freezing and (c) super-quick freezing.

The above results are a comprehensive evaluation of this study. A typical ice-freezing curve is shown in Figure 6. When we started this work, pre-freezing was carried out using a −40 °C refrigerator. As shown in (a) in the figure, the water in the slurry froze slowly, leaving traces of ice crystal growth, and porous bodies with spherical pore shapes like 240-0 could not be prepared. Pre-freezing with liquid nitrogen was, therefore, considered for rapid freezing to inhibit ice crystal growth. In (b), the temperature of the central part of the sample did not decrease via pre-freezing with liquid nitrogen alone, as shown in 0-10, 20-10, and 30-10, and only the central part was frozen in the −40 °C freezer, leaving traces of ice crystal growth in the central part. As shown in (c) in the diagram, super-quick-freezing conditions such as 10-20, 20-20, and 30-20 were necessary for the pre-freezing, where ice growth was suppressed to the center of the specimen. In other words, the key point of the freezing technique in this work is how quickly the temperature range of −1 to −5 °C, the maximum ice crystal formation zone [41], can be progressed and frozen.

Furthermore, this super-quick-freezing technique may lead to brittleness and damage of the frozen samples via over-immersion in liquid nitrogen, but we consider that the CNF added as a foaming stabilizer also has a reinforcing effect on the mechanical strength of the frozen samples.

Table 1 summarizes the pore circularity values calculated from the SEM images of the 30-20 sample and the heat-dried material. Note that the pore circularity increases as the values approach 1.0. The values for the samples made using heat-drying significantly differed, with an average of 0.85. In contrast, the 30-20 sample produced using freeze-drying showed no difference in pore circularity between different sites, with an average value of 0.97. These data confirm that improved circularity was obtained relative to the heat-dried sample. In addition, the pore circularity of the other samples obtained using the freeze-drying method was also measured, and the values ranged from 0.97 to 0.98, as shown in 30-20.

**Figure 7:**
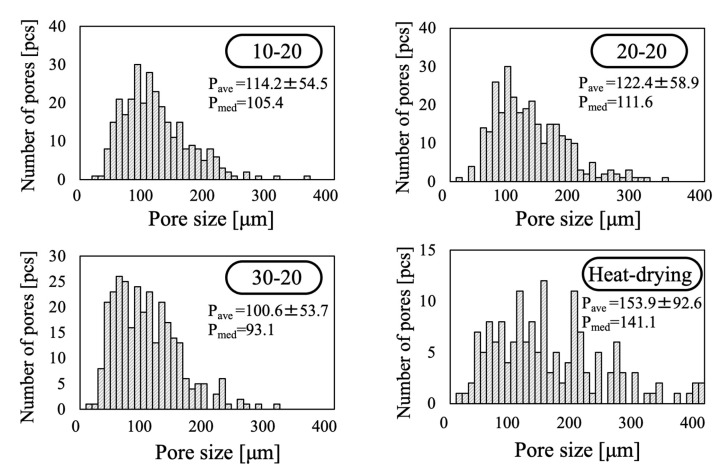
Pore size histograms for various porous β-TCP specimens.

Figure 7 provides pore diameter histograms obtained from analysis of SEM images of the 10-20, 20-20, 30-20, and heat-drying samples. Here, the horizontal axis represents the pore diameter while the vertical axis indicates the number of pores. These data confirm that the pore diameters for all samples were distributed over a wide range from 50 to 200 µm. The mean ± SD and median pore sizes for the samples immersed in liquid nitrogen were all approximately 100 µm, while the values for the heat-drying sample were drastically increased. This discrepancy is attributed to the fact that cracked pores in the specimens disrupted the pore shapes and also indicates an effect whereby pores smaller than 50 µm were very difficult to observe. It is also apparent that the pore sizes for the foams did not change when the freezing conditions were changed, presumably because the foams were frozen instantaneously.

**Figure 8:**
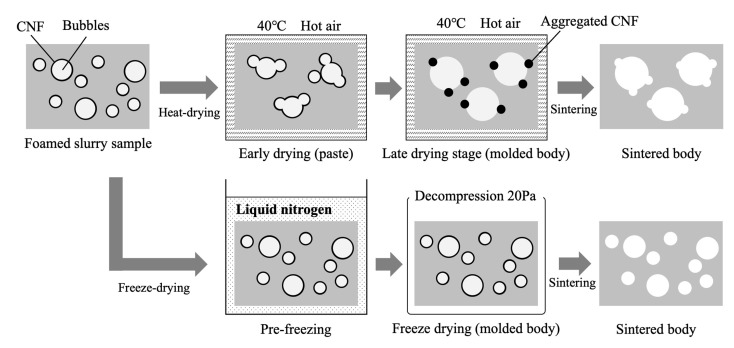
Schematic illustration showing pore formation in foams using different drying methods.

The formation of pores within these materials, using the different drying methods, is illustrated in Figure 8. Drying with hot air required a longer time span such that bubbles had more time to undergo coalescence. During sintering, the CNFs were burnt off and produced pores so the samples generated using heat-drying were more likely to have distorted sphere-like pores. In contrast, freeze-drying after instantaneous pre-freezing prevented the coalescence of bubbles, resulting in the formation of many pores with smaller diameters. The freezing of the CNFs while they were dispersed in the slurry prevented their agglomeration, providing more spherical pores [35].

**Figure 9:**
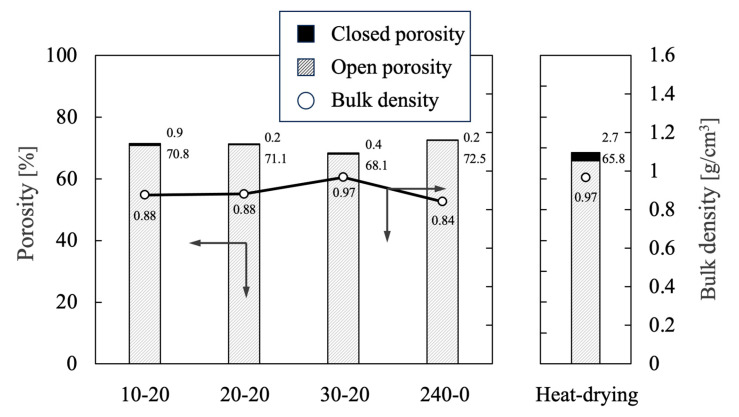
Bulk density and porosity values for porous β-TCP specimens as determined using the Archimedes method. The directions of the arrows indicate the relationship between the axes in the figure. The numerical values for porosity indicate closed porosity in the upper part and open porosity in the lower part. Data on heat-drying were sourced from reference [35].

Figure 9 summarizes the open porosity, closed porosity, and bulk density values for each sample as ascertained using the Archimedes method. The open porosity for each of the freeze-dried specimens was approximately 70%, indicating that highly porous materials were prepared. The closed porosity values for all samples were less than 1% of the total porosities, demonstrating that these bodies should be more permeable than those prepared using heat-drying. Freeze-drying evidently increased pore connectivity because the CNFs were dispersed throughout each sample and generated pores as they were removed during sintering [35].

We investigated the dimensional changes of the samples obtained throughout the preparation process. No significant shrinkage was observed in the freeze-dried samples after drying, but a 35–37% shrinkage was observed in the samples after sintering. On the other hand, even for the heat-dried specimens, a shrinkage of 30–33% was observed in the specimens after sintering.

**Figure 10:**
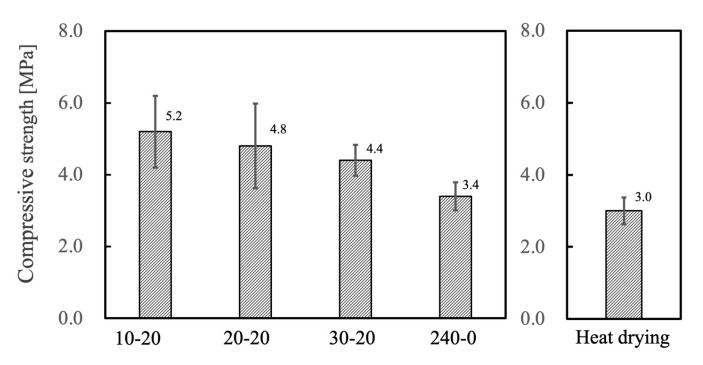
Compressive strength for porous β-TCP specimens fabricated in this work. Data on heat-drying were sourced from reference [35].

The compressive strength test results for all samples are shown in Figure 10. The compressive strength of each freeze-dried product exceeds 2 MPa, indicating that sufficient mechanical strength has been achieved to withstand handling. There was a correlation between the porosity of each specimen and its compressive strength, such that more porous materials had lower compressive strength. The 240-0 sample had the lowest compressive strength, which was assumed to be due to the crack-like pores observed in the SEM images. The specimens made using heat-drying had distorted sphere-like pores and, therefore, exhibited reduced compressive strength despite their low porosity values. Each of the materials containing only spherical pores was found to exhibit both high porosity and good compressive strength, indicating that spherical pores promoted both mechanical strength and porosity.

**Figure 11:**
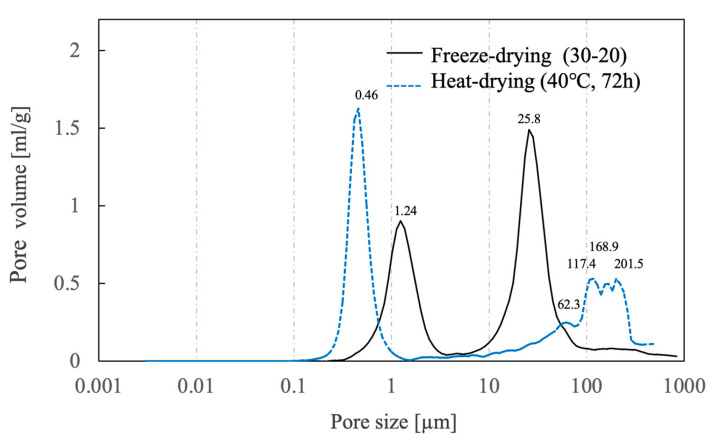
Pore distributions for porous β-TCP specimens fabricated using different drying methods as determined with the mercury injection method. Data on heat-drying were sourced from reference [35].

Figure 11 shows the results of pore distribution measurements using the mercury injection method for samples immersed in liquid nitrogen for 30 s and immersed for 20 s on freeze-dried or heat-dried [35]. The heat-dried sample was found to contain micropores with a maximum pore size of 0.46 ± 0.08 µm and a wide range of macropores with sizes of 50–200 µm. The freeze-dried samples exhibited micropores with a maximum pore size of 1.24 ± 0.25 µm and macropores with a maximum pore size of 25.8 ± 4.7 µm in the 5–80 µm range. These data indicate a transition of the micropores to larger sizes and of the macropores to smaller sizes compared with the heat-dried samples. The micropores likely became larger due to the absence of shrinkage in the case of the freeze-dried materials, as the pore shapes changed only minimally when the specimens were dried while frozen. The macropores are thought to have shifted to smaller sizes because bubbles did not coalesce during drying. These data also suggest that the pores in these materials were connected to the exterior surfaces.

**Figure 12:**
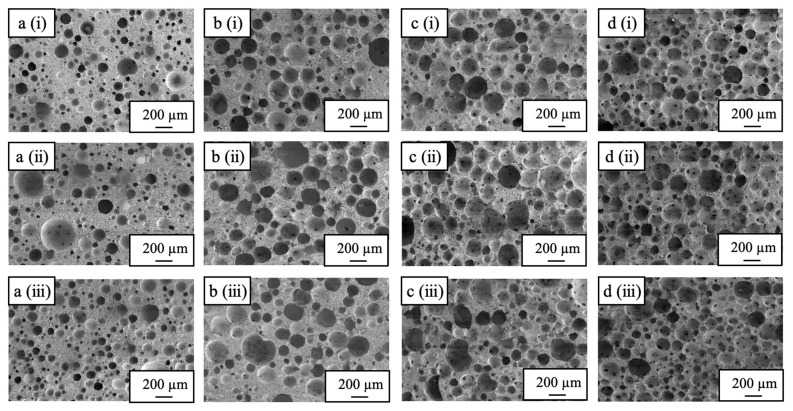
SEM images of cross-sections of porous β-TCP specimens fabricated using nonionic surfactants (**a**) BT-5, (**b**) BT-7, (**c**) BT-9, and (**d**) BT-12, showing (**i**) top, (**ii**) center, and (**iii**) bottom sections (Secondary electron detector, high vacuum mode, accelerated voltage 5 kV).

In additional trials, the HLB values [39] for the nonionic surfactants used as foaming agents were varied. The HLB value is an indicator of the hydrophobicity/hydrophilicity of each surfactant, with a higher value reflecting a more hydrophilic compound. A nonionic surfactant with a low HLB value typically shows reduced foaming properties and generates smaller pores. Cross-sectional SEM images of porous β-TCP bodies prepared using nonionic surfactants with different HLB values are shown in Figure 12. Pores less than 50 µm in size were predominant in the sample made with the BT-5 (having an HLB of 10.5) while larger pores were obtained when using the BT-7 (HLB 12.0), BT-9 (HLB 13.5), and BT-12 (HLB 14.5). Materials with more macro-sized pores having larger pore diameters were obtained using foaming agents with higher HLB values.

**Figure 13:**
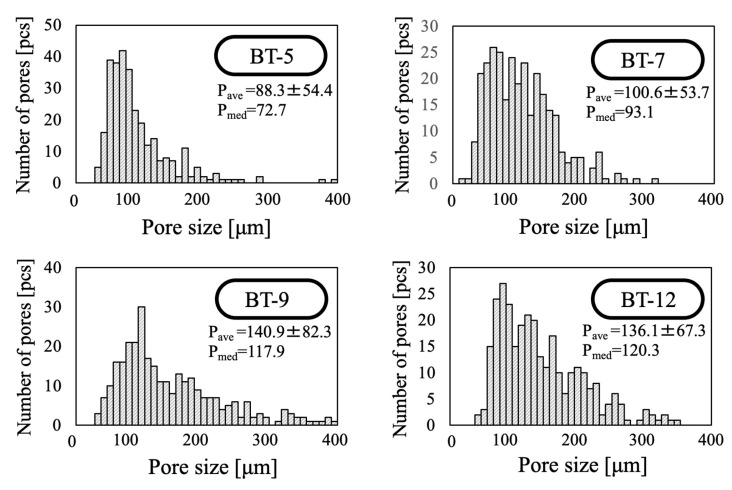
Pore size histograms for porous β-TCP specimens fabricated using different nonionic surfactants.

Figure 13 provides pore size histograms for each sample as obtained through the analysis of the SEM images. The average pore size ± SD and median diameter in the BT-5 sample were 88 ± 54 and 73 μm, respectively; the values obtained with the BT-7 were 100 ± 54 and 93 μm, respectively; those with the BT-9 were 140 ± 83 and 128 μm, respectively; and those with the BT-12 were 136 ± 67 and 120 μm, respectively. These results show that the addition of foaming agents with high HLB values increased both the average pore size and the median diameter. In particular, the use of the BT-9 or BT-12 came close to producing saturation and gave macro-sized pore diameters of more than 180 µm.

**Figure 14:**
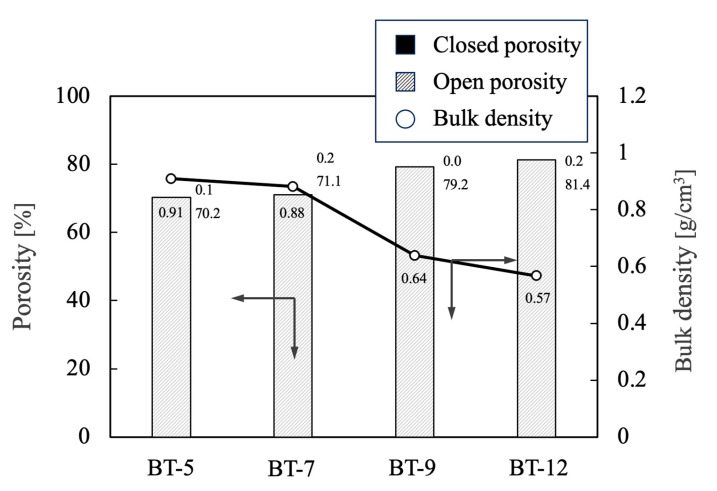
Bulk density and porosity values for porous β-TCP specimens fabricated using different nonionic surfactants. The directions of the arrows indicate the relationship between the axes in the figure. The numerical values for porosity indicate closed porosity in the upper part and open porosity in the lower part.

The bulk densities and porosities of the porous materials prepared by adding foaming agents with different HLB values are summarized in Figure 14. The bulk density and porosity values obtained with the BT-5 and BT-7 were approximately 0.9 g/cm^3^, along with open porosity values of 70%. However, the BT-9 and BT-12 provided values of approximately 0.6 g/cm^3^ with open porosities of 80%. In both cases, the closed porosity of the sample was almost 0%.

**Figure 15:**
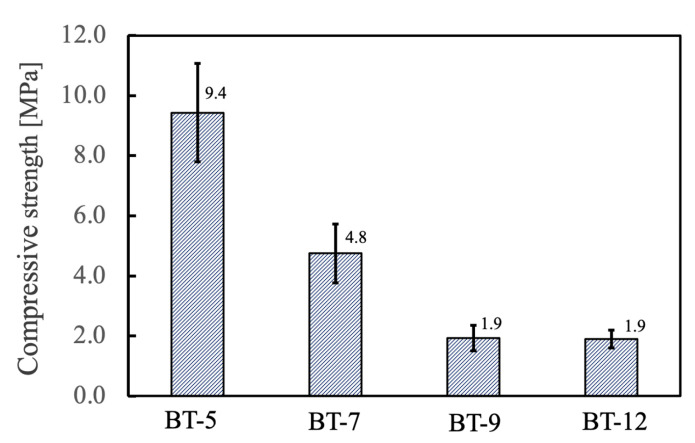
Compressive strength of porous β-TCP specimens fabricated using different nonionic surfactants.

The compressive strengths of the various materials are provided in Figure 15. The compressive strengths of the specimens evidently decreased with the use of foaming agents having higher HLB values. The BT-9 and BT-12, both of which provided greater porosity, produced materials with strengths on the order of 1.9 MPa. Based on the above results, it appears that a foaming agent with an HLB value intermediate between those of BT-7 and BT-9 would be ideal and could produce materials simultaneously exhibiting both high porosity and good compressive strength.

**Figure 16:**
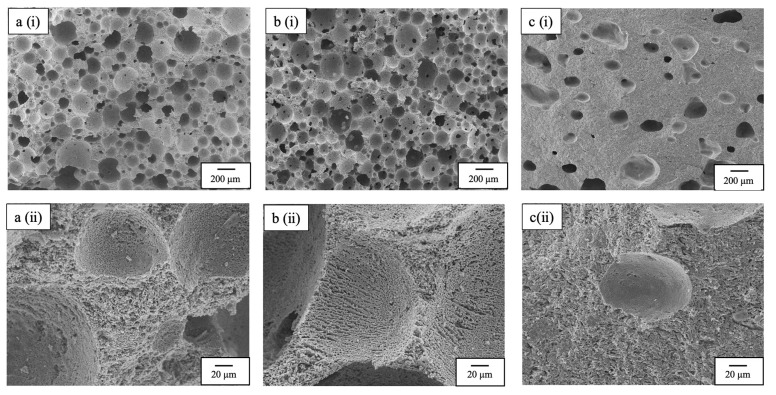
SEM images of fracture surfaces of specimens broken by mechanical stress; (**a**) 30-20, (**b**) BT-12, and (**c**) heat-dried specimens; (**i**) magnification of 50× and (**ii**) 500× (Secondary electron detector, high vacuum mode, accelerated voltage 5 kV).

SEM images of the fracture surface of a specimen broken by mechanical stress are shown in Figure 16. The samples selected were 30-20 with high mechanical strength, BT-12 with high porosity, and heat-dried. The stress direction is from the top of the SEM image. The fracture surfaces of the 30-20 and BT-12 samples show that the thin part of the pillar constituting the porous material has been broken (probably intergranular fracture). The SEM images at high magnification also show that the freeze-dried samples are all composed of continuous pores as small as 1 µm. On the other hand, the heat-dried sample shows both intergranular and intragranular fracture (probably brittle fracture), and the SEM image at high magnification shows that the pores are smaller than the pore size of the freeze-dried sample. These micro-sized continuous pore sizes supported the results obtained using the mercury injection method already described.

Finally, this current study has shown that freeze-drying requires a technique that minimizes ice crystal growth as much as possible in the pre-freezing stage, but the pre-freezing conditions would change if the whole size and shape of the sample were to change. The pore size of the obtained porous materials could not be significantly controlled by changing the pre-freezing conditions, but this could be controlled by changing the slurry viscosity. Additionally, the current study focused on the physicochemical evaluation of the obtained porous materials, but in vitro evaluation using bone-like cells and in vivo animal experiments will be the subject of future work.

## 3. Materials and Methods

### 3.1. Preparation of Raw Powder

In this procedure, 11.32 g of calcium carbonate (CaCO_3_, purity 99.0%, Fujifilm Wako Pure Chemicals, Osaka, Japan) and 39.51 g of calcium hydrogen phosphate dihydrate (CaHPO_4_·2H_2_O, purity 99.5%, Fujifilm Wako Pure Chemicals, Osaka, Japan) were mixed in a Ca/P molar ratio of 1.50. This mixture was subsequently combined with 450 mL of pure water heated to 80 °C, and then ground using zirconia balls (Nikkato Corporation, Osaka, Japan; YSZ balls, 400 g) in a zirconia pot (As One Corporation, Osaka, Japan), having dimensions of 100 mm (width) × 130 mm (height) for 24 h. Each mixed sample was then spread in a plastic container and dried at 70 °C for 24 h. Following this, each dried specimen was ground using an agate mortar and then calcined in an electric furnace by heating it to 750 °C at 3 °C/min with a hold time of 10 h. The calcined powders were identified using X-ray diffraction (XRD, MiniFlex 600: Rigaku Corporation, Tokyo, Japan), and samples diluted in KBr were measured for IR spectra using a Fourier-transform infrared (FT-IR) spectrometer (FT-IR-4200: Japan Spectroscopic Corporation, Tokyo, Japan) equipped with a diffuse reflection device.

### 3.2. Preparation of Porous Materials via Freeze-Drying

**Figure 17:**
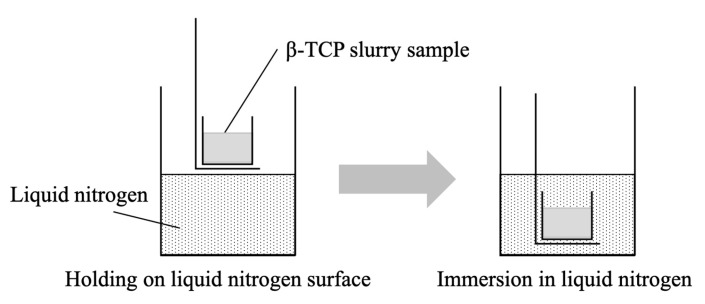
Schematic illustration showing the pre-freezing process.

In each trial, 30 g of the calcined powder was mixed with 30 g of a 3.0 wt% dispersion of CNFs in water (fiber width: 0.069 µm, fiber length: 9.66 µm: Daio Paper Corporation, Shikoku, Japan) together with 30 mL of ammonium polyacrylate solution 70–110 (molecular weight: approximately 10,000, Fujifilm Wako Pure Chemicals, Osaka, Japan), diluted to 5 vol% and dispersed in cold water using a hand blender (HB-502WJ: Cuisinart, Tokyo, Japan) in an ultrasonication device (As One, Tokyo, Japan) filled with ice water for 5 min. Subsequently, a 4 mL quantity of a polyoxyethylene alkyl ether nonionic surfactant (BT-7, Nikko Chemicals Corporation, Tokyo, Japan) was added as a foaming agent. Foaming was then carried out with mechanical agitation under the same conditions as used to produce the original dispersions. The resulting foam was tapped at a height of approximately 20 mm and poured into a polypropylene mold (25 mm in length × 25 mm in width × 25 mm in height). Each specimen was subsequently frozen by being suspended above the surface of liquid nitrogen in a stainless-steel dewar (at which location the ambient temperature was −180 °C), as shown in Figure 17. The sample was then pre-frozen via direct immersion in the liquid nitrogen (at a temperature of −196 °C) and then stored in a −40 °C freezer. The temperatures for these processes were monitored by temperature sensors for cryogenic use. Following this, each pre-frozen sample was freeze-dried using an FDU-1200 apparatus (EYELA, Tokyo, Japan) at −40 °C and 20 Pa. Each of the dried specimens was then sintered in an electric furnace by heating at a rate of 5 °C/min to 180 °C and holding at that temperature for 4 h followed by a 4 h hold at 300 °C, a 4 h hold at 400 °C, and a 4 h hold at 1100 °C. The sintering program was determined based on the results of the thermal analysis of dried samples (TG-DTA instrument; Thermo Plus Evo 2, TG-DTA-8122, Rigaku Corporation, Tokyo, Japan).

The time spans over which each specimen was held above the liquid nitrogen surface and the immersion times in the liquid nitrogen are given in Table 2. Note that each sample is referred to herein using the notation x-y, where x is the hold time [s] above the liquid nitrogen and y is the immersion time [s] in the liquid nitrogen. To allow for a comparison of drying methods, the samples were also prepared via heat-drying at 40 °C for 72 h using an NDO-450ND apparatus (EYELA, Tokyo, Japan), after which the same sintering program was applied.

### 3.3. Preparation of Porous Materials Using Nonionic Surfactants with Different HLB Values

In these procedures, 30 mL of a 5 wt% ammonium polyacrylate solution, 30 g of a 3.0 wt% dispersion of CNFs in water, and 30 g of the calcined β-TCP powder were mixed for 5 min using a hand mixer in an ultrasonication device filled with ice water. Following this, 4 mL of a polyoxyethylene alkyl ether surfactant (either BT-5, BT-7, BT-9, or BT-12, Nikko Chemicals Corporation, Tokyo, Japan) was added as a foaming agent and the specimen was foamed for 5 min. After tapping, the mixture was poured into a polypropylene mold and first frozen through holding for 30 s above the surface of liquid nitrogen then pre-frozen through direct immersion for 20 s in the liquid nitrogen. Samples were subsequently stored in a freezer. Following this, each frozen specimen was freeze-dried at −40 °C and 20 Pa. The freezing conditions were 30-20 using the notation described above. The dried samples were sintered using the method described in the previous section. The HLB values of the foaming agents used in this study were 10.5, 12.0, 13.5, and 14.5 for the BT-5, BT-7, BT-9, and BT-12, respectively [39].

### 3.4. Evaluation of Porous Materials

Some of the porous materials were crushed and the resulting powders were characterized using XRD and FT-IR spectroscopy. The samples were also cut into sections along the vertical direction and the pore structures of these sections were evaluated using scanning electron microscopy (SEM, JSM-7000, JEOL Ltd., Tokyo, Japan). Samples for SEM were gold-coated using magnetron sputtering equipment (MSP-1S, Vacuum Device Inc., Ibaraki, Japan). These sections comprised the top, center, and bottom parts with respect to the height direction of each sample. From the SEM images, the largest diameters of 300 randomly selected pores were determined for each specimen using the ImageJ image processing software (https://imagej.net/ij/, accessed on 20 April 2022) The data obtained were statistically processed on Excel sheets and expressed as means ± standard deviation. In addition, 30 pores were randomly selected from SEM images of the top, center, and bottom sections of each specimen, and the areas and perimeters of these pores were ascertained. The corresponding circularity value for each pore (having a value between 0 and 1) was then calculated as

circularity = 4π [area]/[perimeter]^2^ (1)

The porosity and bulk density of each material were determined using the Archimedes method, following the procedure in the JIS R 1634 standard [42]. Compressive strength tests based on the JIS R 1608:2003 standard [43] were carried out using a universal testing machine (Little Senstar, Tokyo Testing Machinery, Tokyo, Japan). The six specimens used for the compressive strength test were cut to 4 mm (length) × 4 mm (width) × 10 mm (height) and the cut surfaces were not polished. The data obtained were statistically processed on Excel sheets and expressed as means ± standard deviation. Pore size distributions and pore volumes were obtained using the mercury injection method, employing an Autopore IV 9520 instrument (Shimadzu Corporation, Kyoto, Japan). The specimen used for this test was cut to 10 mm (length) × 10 mm (width) × 5 mm (height) and the cut surfaces were not polished.

## 4. Conclusions

The use of freeze-drying to prepare porous materials could significantly promote the formation of spherical pores to provide materials with both high compressive strength and good porosity. Such materials also exhibited exceptional permeability. Holding specimens above liquid nitrogen for several tens of seconds prior to immersion in liquid nitrogen suppressed shape changes due to ice crystal growth and a short (20 s) immersion in liquid nitrogen was found to be sufficient to freeze the samples. The specimens obtained using freeze-drying were found to be porous with a wide pore size distribution in the 50–200 µm range and had mean and median pore sizes of approximately 100 µm for all freeze-drying conditions. Mercury porosimetry results showed that the samples contained continuous micro-sized pores with a maximum pore volume of 1.24 µm and continuous macro-sized pores with a maximum pore volume of 25.8 µm and a pore size distribution of 10–80 µm. Samples made using surfactants having lower HLB values showed smaller pore diameters and reduced porosity while materials obtained using foaming agents with higher HLB values exhibited larger pore diameters with more macro-sized pores. Even so, it was not possible to control the pore size solely by adjusting the HLB value. Materials showing both high porosity (>75%) and suitable compressive strength (>2 MPa) were obtained, and the data suggested that the use of a foaming agent with an HLB value between 12.0 and 13.5 was optimal.

## Figures and Tables

**Table 1:** Pore circularity values for porous β-TCP specimens obtained using different drying methods.

Location	Heat-Drying (40 °C, 72 h)	Freeze-Drying (30-20)
Top	0.87	0.98
Center	0.82	0.97
Bottom	0.86	0.97
Average	0.85	0.97

**Table 2:** Hold times for samples above the liquid nitrogen surface and immersion times in liquid nitrogen.

	Hold Time	0 s	10 s	20 s	30 s	240 s
Immersion Time						
0 s						240-0
10 s		0-10	10-10	20-10	30-10	
20 s		0-20	10-20	20-20	30-20	
30 s		0-30	10-30	20-30	30-30	

## Data Availability

Data are contained within the article.

## References

[1] Kolk A., Handschel J., Drescher W., Rothamel D., Kloss F., Blessmann M., Heiland M., Wolff K.D., Smeets R. (2012). Current trends and future perspectives of bone substitute materials–From space holders to innovative biomaterials. J. Cranio Maxillofac. Surg..

[2] Laurencin C., Khan Y., El-Amin S.F. (2006). Bone graft substitutes. Expert Rev. Med..

[3] Giannoudis P.V., Dinopoulos H., Tsiridis E. (2005). Bone substitutes: An update. Injury.

[4] Bandyopadhyay A., Mitra I., Goodman S.B., Kumar M., Bose S. (2023). Improving biocompatibility for next generation of metallic implants. Pro. Mater. Sci..

[5] Dorozhkin S.V. (2010). Bioceramics of calcium orthophosphates. Biomaterials.

[6] Bignon A., Chouteau J., Chevalier J., Fantozzi G., Carret J.-P., Chavassieux P., Boivin G., Melin M., Hartmann D. (2003). Effect of micro- and macroporosity of bone substitutes on their mechanical properties and cellular response. J. Mater. Sci. Mater. Med..

[7] Bohner M., Santoni B.L.G., Döbelin N. (2020). β-tricalcium phosphate for bone substitution: Synthesis and properties. Acta Biomater..

[8] Owen G.R., Dard M., Larjava H. (2018). Hydoxyapatite/beta-tricalcium phosphate biphasic ceramics asregenerative material for the repair of complex bone defects. J. Biomed. Mater. Res. Part B Appl. Biomater..

[9] Legeros R.Z., Lin S., Rohanizadeh R., Mijares D., Legeros J.P. (2003). Biphasic calcium phosphate bioceramics: Preparation, properties and applications. J. Mater. Sci. Mater. Med..

[10] Bouler J.M., Pilet P., Gauthier O., Verron E. (2017). Biphasic calcium phosphate ceramics for bone reconstruction: A review of biological response. Acta Biomater..

[11] Linhart W., Briem D., Amling M., Rueger J.M., Windolf J. (2004). Mechanical failure of porous hydroxyapatite ceramics 7.5 years after implantation in the proximal tibial. Unfallchirurg.

[12] Ogose A., Hotta T., Kawashima H., Kondo N., Gu W., Kamura T., Endo N. (2005). Comparison of hydroxyapatite and beta tricalcium phosphate as bone substitutes after excision of bone tumors. J. Biomed. Mater. Res. Part B Appl. Biomater..

[13] Shimazaki K., Mooney V. (1985). Comparative study of porous hydroxyapatite and tricalcium phosphate as bone substitute. J. Ortho. Res..

[14] Choi Y.J., Chang H.J., Kim M.J., Lee L.H., Lee B.K. (2023). Efficacy of pure beta tricalcium phosphate graft in dentoalveolar surgery: A retrospective evaluation based on serial radiographic images. Maxillofac. Plast. Reconstr. Surg..

[15] Bohner M., Baroud G., Bernstein A., Döbelin N., Galea L., Hesse B., Heuberger R., Meille S., Michel P., von Rechenberg B. (2017). Characterization and distribution of mechanically competent mineralized tissue in micropores of β-tricalcium phosphate bone substitutes. Mater. Today.

[16] Bohner M., Lemaitre J. (2009). Can bioactivity be tested in vitro with SBF solution?. Biomaterials.

[17] Kondo N., Ogose A., Tokunaga K., Ito T., Arai K., Kudo N., Inoue H., Irie H., Endo N. (2005). Bone formation and resorption of highly purified β-tricalcium phosphate in the rat femoral condyle. Biomaterials.

[18] Yuan H., Fernandes H., Habibovic P., de Boer J., Barradas A.M.C., de Ruiter A., Walsh W.R., van Blitterswijk C.A., de Bruijn J.D. (2010). Osteoinductive ceramics as a synthetic alternative to autologous bone grafting. Proc. Natl. Acad. Sci. USA.

[19] Dong J., Uemura T., Shirasaki Y., Tateishi T. (2002). Promotion of bone formation using highly pure porous β-TCP combined with bone marrow-derived osteoprogenitor cells. Biomaterials.

[20] Bohner M., Baumgart F. (2004). Theoretical model to determine the effects of geometrical factors on the resorption of calcium phosphate bone substitutes. Biomaterials.

[21] Mayr H.O., Klehm J., Schwan S., Hube R., Sudkamp N.P., Niemeyer P., Salzmann G., von Eisenhardt-Rothe R., Heilmann A., Bohner M. (2013). Micropourous calcium phosphate ceramics as tissue engineering scaffolds for the repair of osteochondral defect: Biomechanical results. Acta Biomater..

[22] Huang B., Yang M., Kou Y., Jiang B. (2024). Absorbable implants in sport medicine and arthroscopic surgery: A narrative review of recent development. Bioact. Mater..

[23] Levengood S.K.L., Polak S.J., Wheeler M.B., Maki A.J., Clark S.G., Jamison R.D., Johnson A.J.W. (2010). Multiscale osteointegration as a new paradigm for the design of calcium phosphate scaffolds for bone regeneration. Biomaterials.

[24] Wang X., Lin M., Kang Y. (2019). Engineering Porous β-Tricalcium Phosphate (β-TCP) Scaffolds with Multiple Channels to Promote Cell Migration, Proliferation, and Angiogenesis. ACS Appl. Mater. Interfaces.

[25] Park M., Lee G., Ryu K., Lim W. (2019). Improvement of Bone Formation in Rats with Calvarial Defects by Modulating the Pore Size of Tricalcium Phosphate Scaffolds. Biotechnol. Bioproc..

[26] Smoak M., Hogan K., Kriegh L., Chen C., Terrell L.B., Qureshi A.T., Monroe W.T., Gimble J.M., Hayes D.J. (2015). Modulation of mesenchymal stem cell behavior by nano- and micro-sized β-tricalcium phosphate particles in suspension and composite structutes. J. Nanopart. Res..

[27] Chen Y., Wang J., Zhu X.D., Tang Z.R., Yang X., Tan Y.F., Fan Y.J., Zhang X.D. (2015). Enhanced effect of β-tricalcium phosphate phase on neovascularization of porous calcium phosphate ceramics: In vitro and in vivo evidence. Acta Biomater..

[28] Darus F., Isa R.-M., Mamat N., Jaafar M. (2018). Techniques for fabrication and construction of three-dimensional bioceramic scaffolds: Effect on pores size, porosity and compressive strength. Ceram. Inter..

[29] Larionov D.S., Evdokimov P.V., Filippov Y.Y., Shibaev A.V., Philippova O.E., Shipunov G.A., Shcherbakov I.M., Dubrov V.E., Novoseletskaya E.S., Efimenko A.Y. (2023). Mechanical Properties of Ca_3_(PO_4_)_2_-Based Macroporous Bioceramics. Russ. Metall..

[30] Firouzi M., Nguyen A.V. (2017). The Gibbs-Marangoni stress and non DLVO forces are equally important for modeling bubble coalescence in salt solutions. Colloids Surf. A Physicochem. Eng. Asp..

[31] Wang H., Wei X., Du Y., Wang D. (2019). Effect of water-soluble polymers on the performance of dust-suppression foams: Wettability, surface viscosity and stability. Colloids Surf. A Physicochem. Eng. Asp..

[32] Clint J.H. (1992). Surfactant Aggregation.

[33] Vafaei S., Wen D. (2015). Modification of the Young–Laplace equation and prediction of bubble interface in the presence of nanoparticles. Adv. Colloid Interface Sci..

[34] Dufner L., Oßwald B., Eberspaecher J., Riedel B., Kling C., Kern F., Seidenstuecker M. (2023). Adjustment of Micro- and Macroporosity of ß-TCP Scaffolds Using Solid-Stabilized Foams as Bone Replacement. Bioengineering.

[35] Sasaki H., Shibata H., Hashimoto K. (2020). Fabrication of Porous β-Tricalcium Phosphate Using Cellulose-Nano-Fiber. J. Soc. Inorg. Mater. Jpn..

[36] Mochida R., Shibata H., Hashimoto K. (2022). Preparation and Evaluation of Porous β-Type Tricalcium Phosphate by Physical Foaming Method Using Acetylated Cellulose Nanofibers. J. Soc. Inorg. Mater. Jpn..

[37] Toyota G., Shibata H., Hashimoto K. (2022). Preparation of porous β-tricalcium phosphate by foaming method using cellulose nanofiber with different manufacturing methods as foam stabilizer. Phosphorus Res. Bull..

[38] Hashimoto K., Fukuchi M., Shibata H. (2024). Characterization of Porous β-Tricalcium Phosphate Fabricated by Physical Foaming with a Nonionic Surfactant: Effect of Adding a Thicken. J. Ceram. Soc. Jpn..

[39] Griffin W.C. (1954). Calculation of HLB Values of Non-Ionic Surfactants. J. Soc. Cosmet. Chem..

[40] Gibson I.R., Rehman I., Best S.M., Bonfield W. (2000). Characterization of the transformation from calcium-deficient apatite to β-tricalcium phosphate. J. Mater. Sci. Mater. Med..

[41] Tan M., Mei J., Xie J. (2021). The Formation and Control of Ice Crystal and Its Impact on the Quality of Frozen Aquatic Products: A Review. Crystals.

[42] (1998). Test Methods for Density and Apparent Porosity of Fine Ceramics.

[43] (2003). Testing Methods for Compressive Strength of Fine Ceramics.

